# Results of environmental monitoring at pediatrics isolation ward, tertiary hospital in northern of Thailand

**DOI:** 10.1017/ash.2025.144

**Published:** 2025-09-03

**Authors:** Sutthiphan Thanomphan, Mattana Chaiprasert, Nittaya Wungsudthisomsri

**Affiliations:** 1 Department of Nursing Research DevelopmentNakornping, Chiang Mai Province., Thailand; 2 Department of Pediatrics Patients NursingNakornping Hospital, Chiang Mai Province., Thailand; 3 Department of Pediatrics Patients NursingNakornping Hospital, Chiang Mai Province., Thailand

## Abstract

**Background:** Throughout healthcare, the physical environment presents an important source of pathogens that can cause healthcare associated infections (HAIs) To keep patients safe, hospitals must maintain a clean environment and minimize the presence of pathogens. **Objectives:** 1. To identify through environmental monitoring the level of cleanliness in area of pediatrics isolation ward. 2.To assessments of environmental cleaning practice of environmental service (EVS) staff, and healthcare worker. **Methodology:** This retrospective study was done in pediatrics isolation ward, tertiary hospital. Sample sizes were 1 EVS staff, 3 nurses aid, and 102 environmental sites. Data were collected from database of infection control program between February 29, to March 2, 2024 via Infection control assessment and response (ICAR: tool for assessing cleaning practice of EVS staff, personnel by direct performance observations), visual assessment, and monitored the residual bioburden by adenosine tri phosphate (ATP) tests, and swab culture of the surface. Data were analyzed by using descriptive statistics. **Results:** The results of this study revealed that level of cleanliness in area of ward by ATP test found, contaminated spots were highest (61.76%, 21/34) and clean spots were lowest (38.24%, 13/34), while swab culture method found contaminated spots were higher (55.88%, 19/34), clean spots were lower (44.12%, 15/34), and visual monitoring found contaminated and clean spots were 35.29%, 12/5 and 64.71%, 22/34 respectively. The most of contaminated sites were bed rails, toilet sink, Treatment and IV care car, door handle, mop, Light scope & blade (5.88%, 6/102). As the results proved the most organisms were *Acinetobacter spp*., *Escherichia coli*, and *Pseudomonas spp*., respectively. **Conclusion:** This study suggests that the environmental cleaning in specialized area must be monitored continuous with standard methods. It is necessary to promote education and training staffs follow update practice guidelines, especially the participation of disciplinary team motivated effective activities in reducing the microbial contamination.

